# Covid-19 Dataset: Worldwide spread log including countries first case and first death

**DOI:** 10.1016/j.dib.2020.106173

**Published:** 2020-08-17

**Authors:** Hasmot Ali, Md. Fahad Hossain, Md. Mehedi Hasan, Sheikh Abujar

**Affiliations:** Department of Computer Science and Engineering. Daffodil International University, 4/2 Daffodil Tower, Dhaka 1207, Bangladesh

**Keywords:** COVID-19, Pandemic, Data Statistics, Situation Prediction, Spread Chain

## Abstract

The article represents coronavirus spread log history. The duration, coronavirus takes to spread from one country to another country, could be seen in this dataset and could predicted the same for future pandemics through this dataset. It is highly dependent on the cabalistic number of variables that is the main navel of these datasets. Information for this dataset is collected from trusted websites, local and international popular newspapers. This coronavirus dataset not only help to track the spreading route of coronavirus but also can be used for predicting the possible spreading route of similar future pandemics. This dataset consists of 186 countries' useful data related to COVID-19 pandemic from November 17, 2019, to May 16, 2020, with 8 unique variables that provide the information of the nature of the spread of COVID-19. The datasets mainly focus on two major fields, first one is First Case which consists of information of Date of First Case(s), Number of confirm Case(s) at First Day, Age of the patient(s) of First Case, Last Visited Country and the other one First Death information consist of Date of First Death and Age of the Patient who died first for every Country mentioning corresponding Continent.This dataset also can perform a bunch of predictions using Machine Learning applications, like -how fast the virus is spreading, affect rate, death rate, death rate and able to represent comparison between other pandemics. Using this dataset, any similar pandemic spreadness could be predicted earlier and necessary precaution measures could be taken.

**Specifications Table**

**Table:** 

Subject	Epidemiology, Health
Specific subject area	Data related to nature, agility, statistics of promptness of COVID-19, prediction analysis related to pandemics, path of spread of pandemic
Type of data	Table
How data were acquired	Local newspaper via Google search
Data format	Raw Spreadsheets (*.xlsx)
Parameters for data collection	We tried to parameterize our data by the above variables. Country, Date of first case, Source of first case(s), Number of the confirmed case(s) at first day, Age of confirmed case(s) at first date, Last Visited Country(s) of Confirmed Case(s), Date of first death(s), Source of first death(s), Age of first death(s).
Description of data collection	We searched for our required data in the mentioned sources and filtered according to the importance of usefulness and information efficiency. We classified the related data from available websites and datasets and sorted them as our required data for presenting clean and useful data. We focus to add every possible important data for maximum usability.
Data source location	Daffodil International University, Dhaka, Bangladesh
Data accessibility	Repository name: Mendeley Direct URL to data: https://data.mendeley.com/datasets/vw427wzzkk/5

**Value of the Data**

Other researchers can use this dataset for statistical analyses to predict the spread chain of the new pandemic in the future and applying Machine Learning Algorithms they can suggest the possible solution of prevention and insight about the new pandemic, and tell people how to fight through this pandemic.

• Researchers can use this data for statistical analyses, scientists can use this dataset to track the path of the spread of this pandemic, the public can use this data to find out the risk of this pandemic for a different age.
• This dataset can be used to keep track of the path of the spread of COVID-19 and its effect. On the other hand, this dataset can be used to predict the spread chain of a new virus, pandemics in the future. Besides, it can be used to understand the threat of pandemic for different ages in the future. This dataset is also important for the action regarding pandemic for government policies.
• This dataset represents quite easily almost everything to track COVID-19 from countries to countries and continents to continents.
• People also can decide pandemic just have a look at this dataset they can understand easily what they should do and do not.

## 1 Data Description

**Fig. 1:**
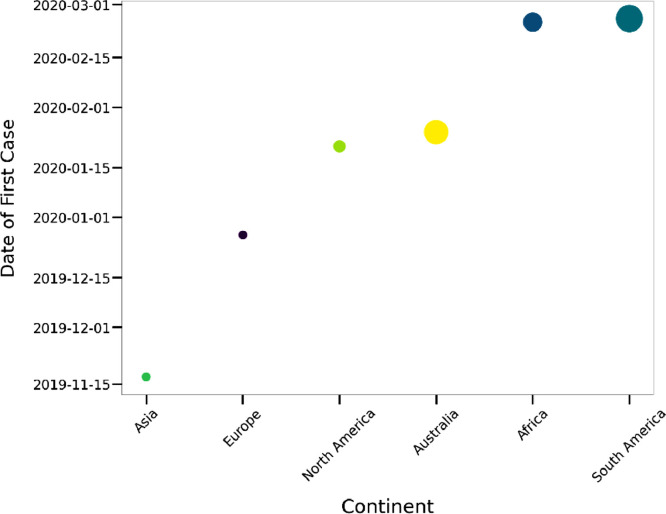
Date of first confirmed case in each continent

Information about the ongoing COVID-19 pandemic has been collected in this dataset. This dataset consists of information from 186 countries. This is a single dataset. There are eight columns with a unique representation of useful data. This dataset stores information about the first confirmed case and first confirmed the death of COVID-19 in 186 countries up to May 16, 2020. Vasilios [1] presents a dataset containing information related to the cluster-based active case, active case per population, and area for every country. Kabir and Peter [2], [3] show the online forecasting mechanism for every 24 h and survey about risk perception and health behavior among Nigerian using Nigerian Government Data. Jing [4] presented a dataset containing 33-death case information from Wuhan for estimating death rate while Marcel [5] presented a combinational dataset of country profile and mobility analysis. Two separate datasets are brought by Cam-Tu [6], [7] about Vietnamese students and teachers' learning habits and perceived support during COVID-19. Toan [8] presents survey data for risk perception and the information related to COVID-19 risk and responsibilities among Italy is published by the Italian Civil Protection Department [9]. But this dataset contains the information about the first confirmed case(s) consists of the date of first confirmed case(s), number of confirmed case(s) at first date, age of confirmed case(s) at first date, last visited country(s) of confirmed case(s). Information about the first confirmed death(s) consists of the date of first confirmed death and age of first confirmed death(s) on the first day. To understand the dataset more clearly, some figures have been given. Fig 1. Shows the date of continent wise first confirmed case. Different size of the circle indicates the difference in count of confirmed cases on the first day.

**Fig. 2:**
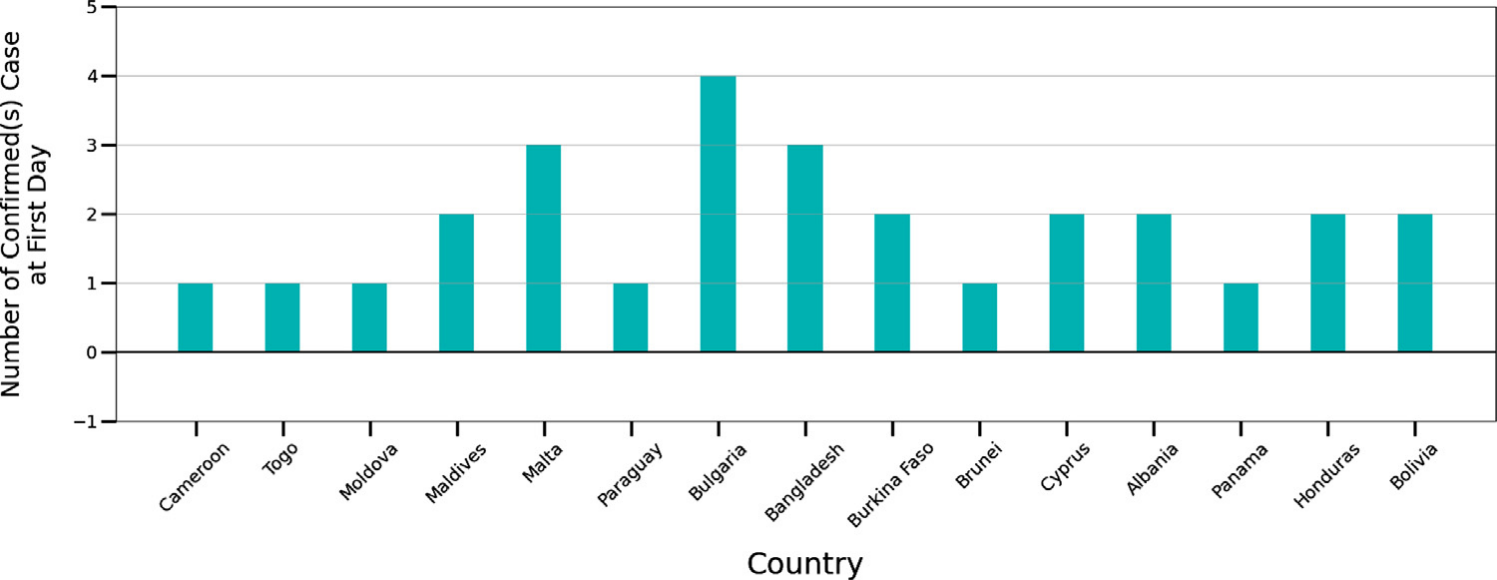
Number of confirmed case(s) at first day.

Fig 2. shows sample data of the number of country-wise confirmed case(s) at first day. In most of countries, only one person was found confirmed on the first day. This data, with some other variables, can be used to predict the number of infections at the first day of any pandemic in the future.

**Fig. 3:**
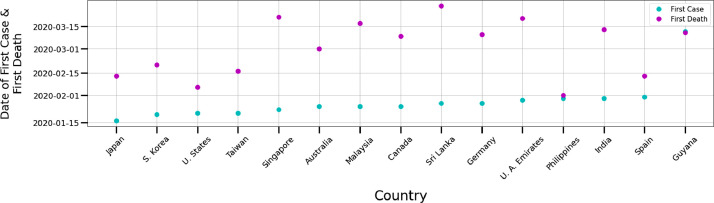
Date of the first confirmed case and first confirmed death of each country.

**Fig. 4:**
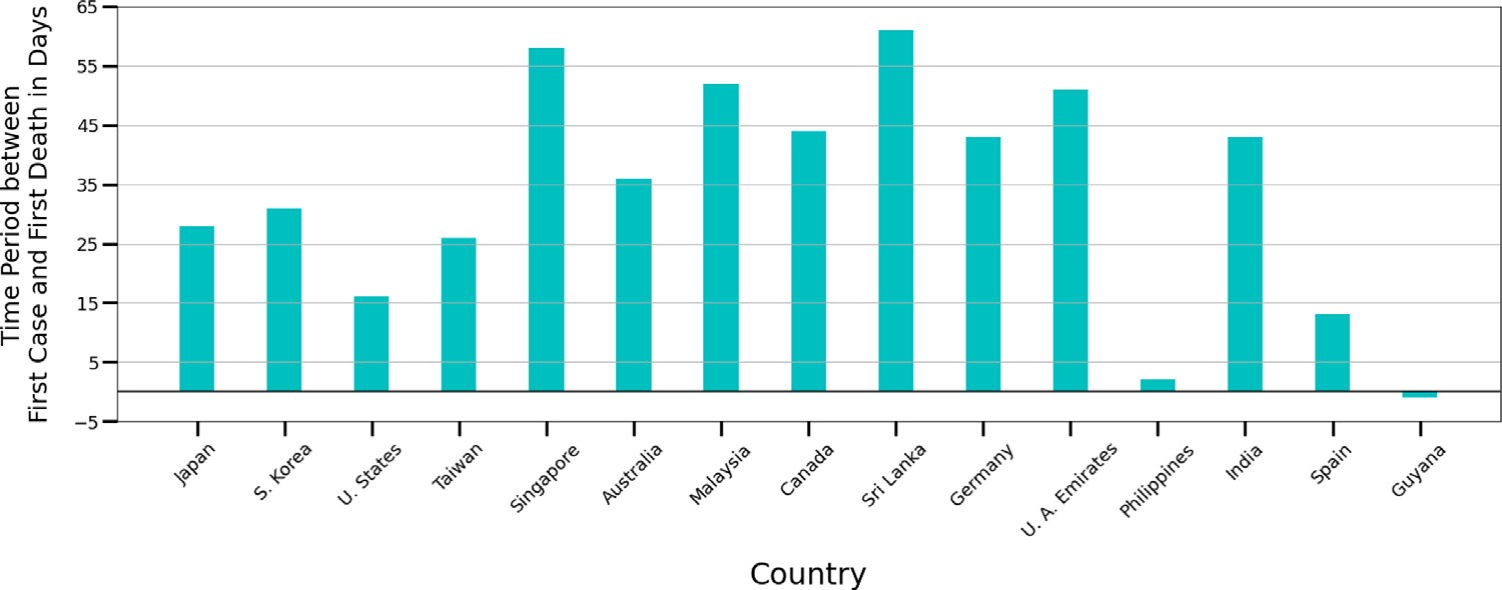
Period between the first confirmed case and first confirmed death in days.

The data shown in Fig. 3, Fig. 4 can be used to estimate the possible date of death after the infection by virus.

Fig 3. shows a sample data of the date of the first confirmed case and the date of the first confirmed death of each country. In some countries, such as Guyana, purple dot (First Death) indicates an earlier date than a cyan dot (First Case). That means the patient died with disease syndrome and the test was completed later.

Fig 4. shows a sample data of the period between the first confirmed case and first confirmed death. Till May 16, 2020, some countries do not have any death cases. Those countries have been ignored in this figure. Some countries have a downward graph which indicates that the patient died before the test result came.

**Fig. 5:**
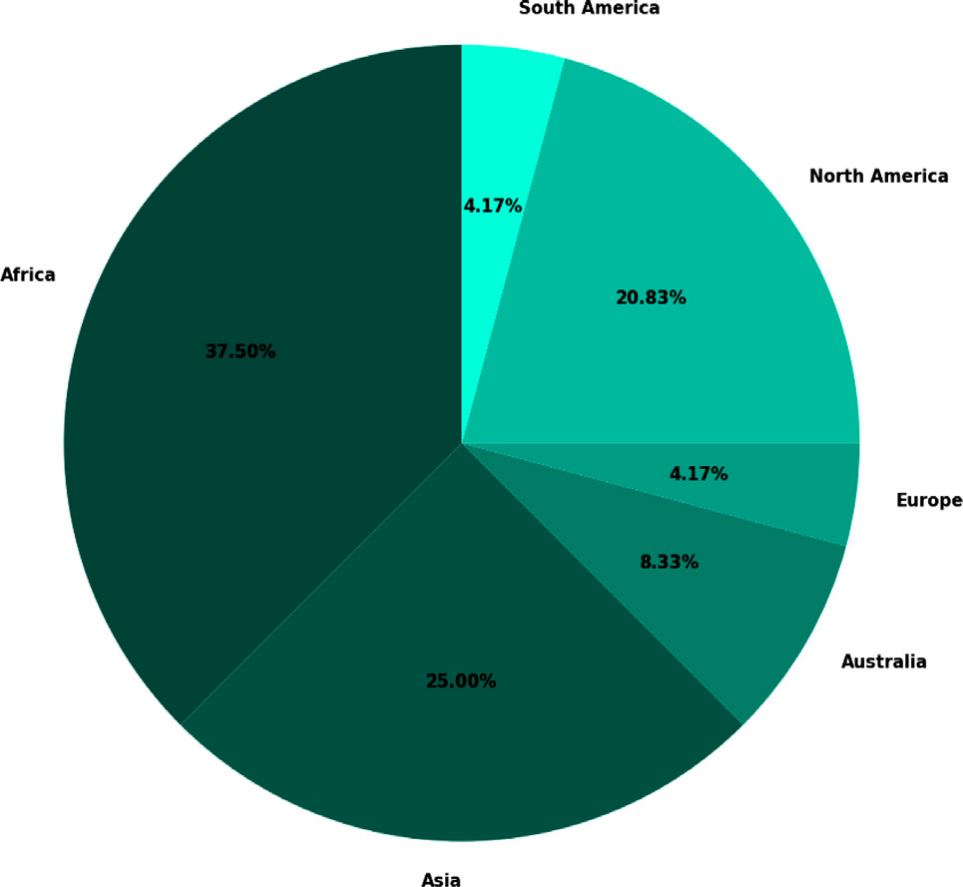
Zero death cases in continents

There is no death case till May 16, 2020, in 24 countries. Fig 5. Gives an idea about the number of countries with zero death cases in each continent as a percentage. 6 months have passed since COVID-19 was first identified. And after 6 months, these regions have no death case and the first confirmed cases were found recently. So, their situation management system can be a field of study to find out a way to prevent the spread of any pandemic.

Table 1 and Table 2 are given to indicate the world widespread chain of COVID-19 pandemic. Both tables are given in a binary matrix. Table 1 shows the spread chain among the continents. Table 2 shows a sample spread chain among countries. The full matrix of Table 2 has been given in our dataset. The spread chain shown in these two tables is the most valuable data of this dataset. These data can be used to track the pandemic in the future. And we can estimate the possible path of travel and date of arrival of the pandemic so that we can take proper preparation to prevent a pandemic.

**Table 1:** Spread of COVID-19 in Continent(s)

	Africa	Asia	Australia	Europe	North America	South America
Asia	0	0	1	0	1	0
Europe	1	0	0	0	0	1

**Table 2:** Spread of COVID-19 in Country(s)

	Colombia	Iraq	Mozambique	Singapore	Togo
Italy	1	0	0	0	0
China	0	0	0	1	0
Iran	0	1	0	0	0
United Kingdom	0	0	1	0	0
France	0	0	0	0	1

In this dataset, some data, indicating age, has been filled in "N+" format, which means the age is not specified but greater than N. Some data was not found which is indicated by “No Trace”, some data was not available from the authority, which is indicated by “Unspecified” and some data was filled with “N/A”. In “Last Visited Country(s) of Confirmed Case(s)” column, “N/A” indicates that the confirmed case(s) of those countries do not have any travel history in recent past; in “Age of First Death(s)” column “N/A” indicates that those countries do not have may death case till May 16, 2020.

## 2 Experimental Design, Materials and Methods

For completing the dataset, we tried to find out how many countries are affected by COVID-19 till May 16, 2020. So, we followed Johns Hopkins Coronavirus Resource Center [10] which provides live updated information about coronavirus. Then we decided our work into two different parts for collecting every important information regarding COVID-19.

## 3 Part 1: First Case of COVID-19

For finding the First Case of every country we follow some fixed question pattern when searching on the web which provides the most suitable information and helps us to find data more efficiently.

Table: Sample question pattern for searching First Case

**Table:** 

first coronavirus case in *country_name
First case of COVID-19 in *country_name
*country_name reported first case coronavirus
*country_name first case of coronavirus
*country_name first case of COVID-19

## 4 Part 2: First Death of COVID-19

For finding the First Death of every country we follow some fixed question pattern when searching on the web which provides the most suitable information and helps us to find data more efficiently.

Table: Sample question pattern for searching First Death

**Table:** 

first death of coronavirus in *country_name
first death of COVID-19 in *country_name
*country_name reported first death of coronavirus
*country_name first death of coronavirus
*country_name first death of COVID-19

After searching from the question pattern we found a lot of website-related to search but we collected our data from these websites which provide most of our required data for a specific country like Continent, Country Name, Date of First Death, Date of first case, Age of both cases, etc in a single website. Then we enter the data into our dataset. We have a complete track of which data is collected from which website and an offline copy of raw data as PDF or JPG format for further investigation. This trace can be found as supplementary material in our dataset.

## Declaration of Competing Interest

The authors declare that they have no known competing financial interests or personal relationships which have, or could be perceived to have, influenced the work reported in this article.

## Appendix Supplementary materials

Image, application 1
